# Exploring the effects of gut microbiota on cholangiocarcinoma progression by patient-derived organoids

**DOI:** 10.1186/s12967-024-06012-x

**Published:** 2025-01-09

**Authors:** Ann-Kathrin Lederer, Nele Görrissen, Tinh Thi Nguyen, Clemens Kreutz, Hannah Rasel, Fabian Bartsch, Hauke Lang, Kristina Endres

**Affiliations:** 1 Department of General, Visceral and Transplantation Surgery, University Medical Center Mainz, 55131 Mainz, Germany; 2 Center for Complementary Medicine, Department of Medicine II, Faculty of Medicine, Medical Center—University of Freiburg, University of Freiburg, 79106 Freiburg, Germany; 3 Department of Psychiatry and Psychotherapy, University Medical Center Mainz, 55131 Mainz, Germany; 4 Institute of Molecular Biology (IMB), 55128 Mainz, Germany; 5 Institute of Medical Biometry and Statistics (IMBI), Faculty of Medicine and Medical Center, 79106 Freiburg, Germany; 6 Faculty of Computer Sciences and Microsystems Technology, University of Applied Sciences Kaiserslautern, 66482 Zweibrücken, Germany

**Keywords:** Bile duct, Biliary tract cancer, Cell culture, Cholangiocarcinoma, Gastrointestinal microbiome, Klatskin, Liver surgery, Neoplasm, Organoids, Tissues

## Abstract

**Background:**

Recent research indicates a role of gut microbiota in development and progression of life-threatening diseases such as cancer. Carcinomas of the biliary ducts, the so-called cholangiocarcinomas, are known for their aggressive tumor biology, implying poor prognosis of affected patients. An impact of the gut microbiota on cholangiocarcinoma development and progression is plausible due to the enterohepatic circulation and is therefore the subject of scientific debate, however evidence is still lacking. This review aimed to discuss the suitability of complex cell culture models to investigate the role of gut microbiota in cholangiocarcinoma progression.

**Main body:**

Clinical research in this area is challenging due to poor comparability of patients and feasibility reasons, which is why translational models are needed to understand the basis of tumor progression in cholangiocarcinoma. A promising approach to investigate the influence of gut microbiota could be an organoid model. Organoids are 3D cell models cultivated in a modifiable and controlled condition, which can be grown from tumor tissue. 3D cell models are able to imitate physiological and pathological processes in the human body and thus contribute to a better understanding of health and disease.

**Conclusion:**

The use of complex cell cultures such as organoids and organoid co-cultures might be powerful and valuable tools to study not only the growth behavior and growth of cholangiocarcinoma cells, but also the interaction with the tumor microenvironment and with components of the gut microbiota.

## Background

Clinical trials are essential for the scientific progress and the development of medical treatment. However, clarifying basic mechanisms through clinical trials is a demanding challenge. The individuality of each patient makes it difficult to compare them with other patients, even if they have the same disease [1]. Furthermore, it is often not possible to recruit large numbers of patients for studies due to feasibility reasons, patient-related barriers or the rarity of the disease [2]. It is therefore of fundamental importance to develop translational models that can serve to investigate the underlying mechanism and relationships, particularly in case of cancer. Translational models are approaches that combine clinical practice and basic science with an interdisciplinary concept. Translational approaches are thus able to integrate different disciplines and expertise as well as their resources and techniques in order to accelerate the scientific progress [3].

Despite scientific progress in recent decades, there are still many malignant diseases, which are associated with a poor prognosis for the patients affected. One of these cancers is the so-called cholangiocarcinoma (CCA), an aggressive malignant tumor of the bile ducts. It is assumed that the gut microbiota could have an effect on the progression of CCA. However, there have only been few reports to date [4]. Based on the aforementioned problem of clinical studies, this work aims to discuss whether translational approaches such as an organoid model could contribute to a better understanding of the relationship between the gastrointestinal microorganisms and the progression of CCA.

## Main text

### The diagnosis of CCA: a death sentence?

The first case series on CCA were published in 1958 by William A. Altemeier and in 1965 by Gerald Klatskin [5, 6]. Altemeier described the course of three patients with a sclerosing carcinoma of the major intrahepatic bile duct, whereas Klatskin reported on 13 patients suffering from adenocarcinoma of the hepatic duct at its bifurcation. The publications by Altemeier and Klatskin underline not only the difficulty of diagnosing and treating CCA, but also the lethality of this tumor entity, as all of their patients died, mostly just a few years after surgery despite maximum therapy. To date, CCA are a rare, but still very deadly and heterogeneous group of tumors that are difficult to classify due to their characteristics [7].

### Classification of CCA

**Fig. 1:**
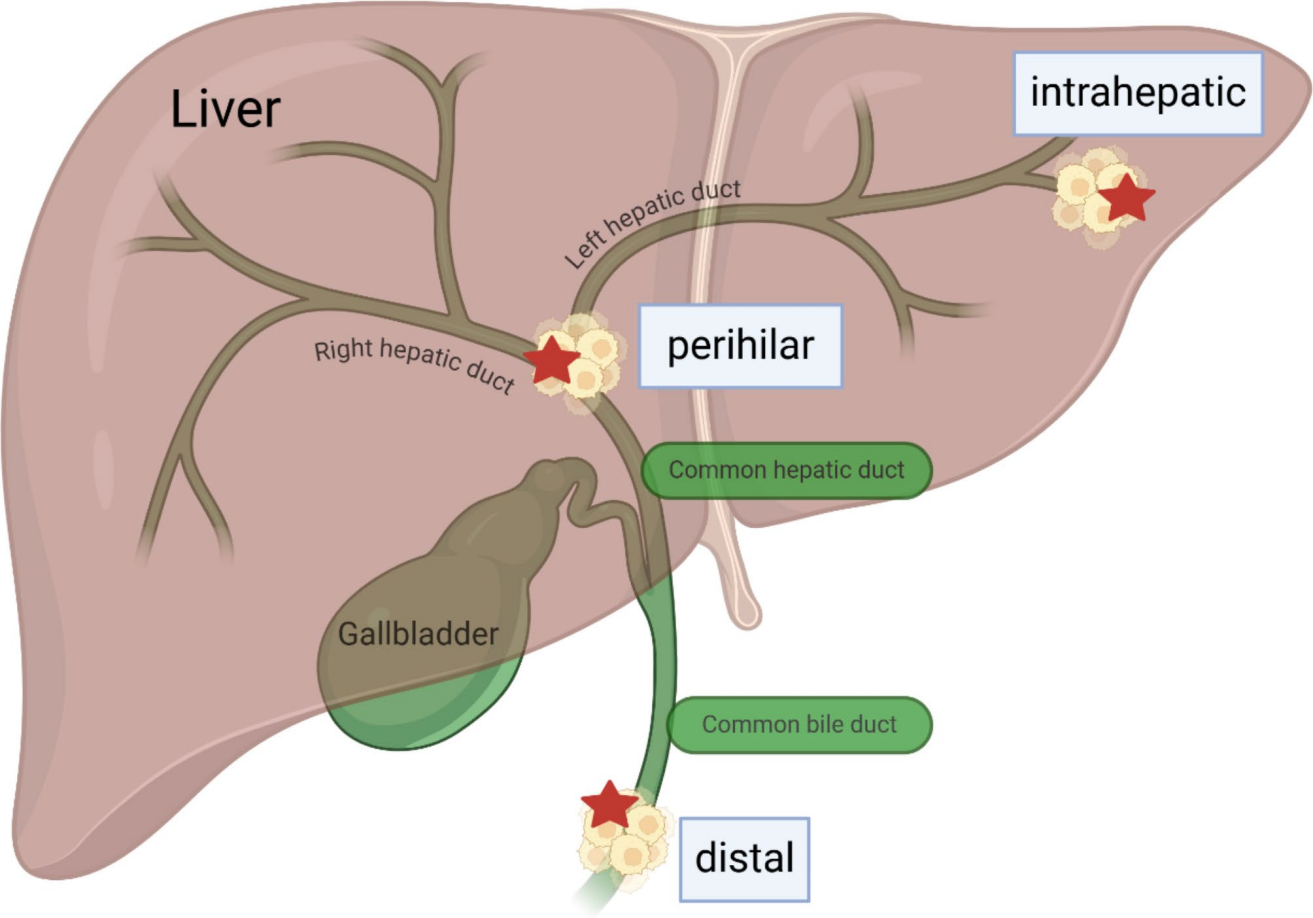
Sites of cholangiocarcinoma (CCA) differentiated in intrahepatic, perihilar (also known as Klatskin) and distal CCA (red stars). Although gallbladder carcinomas are also carcinomas of the biliary system, they are not classified as CCA and must be considered separately due to their different tumor biology; created with Biorender.com)

The CCA is a highly aggressive primary liver tumor. The term CCA covers all carcinomas of the bile ducts and the common bile duct with except of the gallbladder. Gallbladder carcinomas are considered separately due to their different tumor biology [8]. Today, the most commonly used classification for CCA is based on anatomical principles (see Fig. 1).

The frequency of the tumor with regard to its localization is controversially reported. In 2007, DeOliveira et al. retrospectively reported on 564 American patients who underwent surgery between 1973 and 2004 due to CCA. The most frequent tumor localization was perihilar in 50% of patients, followed by a distal localization in 42% of patients [9]. In 2022, Izquierdo-Sanchez et al. reported about 2,234 European patients with a histologically proven diagnosis of CCA made between 2010 and 2019. Contrary to the DeOliveira’s work, the most frequent tumor localization was intrahepatic in 56% of patients [10]. In 2021, Tawarungruang et al. reported on 746 Thai patients with CCA, half of whom suffered from intrahepatic CCA and 39% suffered from perihilar CCA [11]. Walter et al. divided more than 26,000 German patients diagnosed with CCA between 2002 and 2014 into an intrahepatic and an extrahepatic (both perihilar and distal) type [12]. Intrahepatic and extrahepatic CCA occurred with an approximately equal frequency. The authors discussed the previously mentioned differences in the frequency of CCA localization and emphasized that there are several reasons that make it difficult to accurately determine the frequency of CCA localization. For example, inaccurate classification of the tumor can lead to under- or overestimation of tumor types. It must also be assumed that there are regional differences in the frequency of localization, which will be discussed further in section “*Frequency of occurrence and risk factors for the development of CCA*”.

### Clinical symptoms and surgical treatment of CCA

The reason for the usage of an anatomical classification of CCA is that both clinical symptoms of the patients and the surgical therapy depend on the location of the tumor. The early clinical manifestation of CCA is often unspecific or even asymptomatic due to the site of tumor [13, 14]. The first unspecific symptoms may be malaise, loss of appetite or fatigue, which may be followed by weight loss and upper abdominal pain as the disease progresses [7, 13]. Centrally located tumors such as perihilar CCA (also known as Klatskin tumor in memory of Gerald Klatskin [6]) as well as distal located tumors can cause central biliary obstruction leading to cholestasis as well as subsequent jaundice and in case of infection cholangitis [15]. Intrahepatic CCA in particular can become very large without causing specific symptoms as they do not usually cause central biliary obstruction. The therapeutic approach differs, depending on the location of the tumor. Intrahepatic CCA can be treated by liver resection, whereas distal CCA requires pancreatic head resection [16]. The curative resection of perihilar CCA is usually challenging, and major liver surgery is needed, including sometimes also reconstruction of the hepatic artery or portal vein depending on the vascular involvement of the tumor [17, 18]. A critical factor in extended liver resections may be that the residual liver tissue (so-called future liver remnant, FLR) does not ensure adequate liver function leading to liver failure. Thus, in some patients with advanced disease or unfavorable tumor localization it might be necessary to use ALLPS (Associating Liver Partition and Portal vein ligation for Staged hepatectomy), an approach that is only performed by a few experienced liver surgeons [17, 19–21]. In short, ALPPS is a procedure to split the liver in situ in order to achieve hypertrophy of the FLR, which is intended to prevent too little functional liver tissue being available for further life [22]. However, all off these surgical techniques are associated with high morbidity of up to 66% and mortality rates of up to 15% and should therefore only be used at experienced hepatobiliary surgery centers [23, 24].

### Frequency of occurrence and risk factors for the development of CCA

In Germany, the incidence of all types of CCA was around 3–4 per 100 000 inhabitants in 2014, which is similar to the incidence rates of other European countries [12]. The global incidence of CCA varies widely, with particular high rates of 85 per 100 000 inhabitants in northeast Thailand [25]. In general, the incidence rates of CCA are significantly higher in Asia compared to European countries [13, 26]. This differences in incidence probably also reflect local risk factors that favor the development of CCA [25]. Most CCAs arise spontaneously, but CCAs can also occur as a result of pre-existing liver-damaging conditions such as primary sclerosing cholangitis (PSC), bile duct cystic disorders, hepatitis B/C infection, alcohol abuse, all kind of cirrhosis or parasitic infections (for example with *Opisthorchis viverrini* or *Clonorchis sinensis*) [15, 25, 27–29]. Due to the global differences, the risk factors for the development of a CCA must be differentiated. CCA generally affects more men and the elderly [27–29]. In Asia, especially in the poorer parts, infectious diseases are also a major risk factor. Despite the possibility of vaccination, the incidence of hepatitis B in lower-income parts of Asia is more than 10% with a poor chance of treatment [30]. Parasite infections are also endemic in many parts of Asia due to dietary habits and hygiene deficiencies [25, 31]. The liver fluke *Opisthorchis viverrini* is a food-borne infection that affects around 10 million people in Thailand, Laos, Cambodia and southern Vietnam [32]. In addition to infections, the risk of disease and death caused by toxic substances or pollutants is also significantly higher in low-income countries [33]. It is known that exposure to toxic substances such as Thorotrast (radioactive radiocontrast agent used in the past) and asbestos can increase the risk of developing CCA [28, 29, 34]. Besides external influencing factors, there appear to be ethnic differences in incidence and survival of CCA, but it remains unclear whether this differences could also be caused by disease-promoting lifestyle habits and the limited access to quality food and medicine [27]. It is known that lifestyle habits and diet are associated with the composition of the gut microbiota [35]. Thus, these potentially disease-promoting factors could also led to CCA via the gut microbiota. Genetic predispositions appear to play a minor role in the development of CCA, although it should be emphasized that various pre-existing conditions that can lead to CCA have a genetic component [28, 29].

### Histological and molecular biological features of CCA

Histologically, CCAs originate from specialized epithelial cells, the cholangiocytes, which line the biliary ducts [15]. Most of the CCAs are well-to-moderately differentiated, mucin-producing adenocarcinomas [36, 37]. Intrahepatic CCA, in particular, can be further subclassified by their characteristic histological features. Thus, intrahepatic CCA can be divided into a small duct type and a large duct type in accordance to their origin [37]. CCA, especially intrahepatic CCA, can also be differentiated according to their growth behavior, as there are mass-forming (mostly obstructive), intraductal-growing (inside the duct, mostly obstructive) and periductal-growing (along the duct, stenosing) CCA [13]. The histopathological differentiation is of prognostic interest as Bagante et al., for example, reported that intrahepatic CCAs with an intraductal growth pattern are associated with a better prognosis than other growth patterns [38]. There are other classifications, such as the concept of Nakanuma et al., which uses further specific histopathological features of CCA for distinction, but these have currently little influence on the day-to-day decision-making process regarding CCA treatment [37, 39].

Similar to the growth behavior, the molecular patterns of CCA also differ depending on their primary tumor localization and their detailed histological differentiation [26]. Genomic analyses of CCA revealed several mutations, e.g. of the isocitrate dehydrogenase (IDH) 1 and 2 genes, the transformation-related protein 53 (p53) gene and the Kirsten rat sarcoma virus (KRAS) gene as well as an amplification of the epidermal growth factor receptor (EGFR) gene and of the fibroblast growth factors (FGFR) 2 gene, which opens up the possibility of targeted drug therapy [26, 40–42].

### Prognosis

CCA is usually associated with a poor prognosis as the disease progresses or recurs rapidly [36, 43–45]. Due to the late onset of specific symptoms, most of the patients suffer from advanced-stage disease at first presentation [46]. The only curative treatment approach is the complete surgical resection, but only one-third to one-half of patients can be treated surgically due to the advanced stage of the disease at initial diagnosis [10, 19, 47]. In patients suffering from an irresectable tumor, the median survival is reported to be less than one year despite chemotherapy [10]. Novel therapeutic approaches include molecular profiling and targeted drug therapy, which improved the survival of these patients. For example, Goyal et al. investigated the role of futibatinib for FGFR2-rearranged irresectable intrahepatic CCA in a multinational, open-label, single-group, phase 2 study. The median progression-free survival and the median overall survival were 9 months and 22 months, respectively [48]. Furthermore, recent studies indicate that the addition of chemotherapeutical substances such as Cisplatin, and nab-Paclitaxel to the currently predominant chemotherapeutic regimen with gemcitabine could improve the survival of patients with advanced CCA to a median overall survival 19 months [49]. It is known that chemotherapeutic drugs have an influence on the composition of the gut microbiota, but the role of these alteration in cancer progression are widely unclear [50, 51]. However, it is assumed that the microbial composition has an influence on the efficacy and toxicity of chemotherapy [52].

Of all patients who have undergone curative tumor resection, only one third managed to survive for 5 years [46, 53]. Usually, curative therapeutic approaches combine hepatobiliary surgery and systemic chemotherapy [44, 54–56]. In recent years, there has been much discussion about the use of neoadjuvant chemotherapy for CCA. Oncologists concerned that neoadjuvant pretreatment could close the window for a possible resection in some patients, as tumor progression occurs during chemotherapeutic treatment. In fact, the Phase II NEO-GAP trial revealed that almost one fourth of initially resectable and neoadjuvant treated intrahepatic CCA patients had progressed to irresectability or to metastatic disease [57]. Nevertheless, a systematic review of retrospectively collected or neoadjuvant pre-treated patients with primary irresectability showed that neoadjuvant chemotherapy could have an advantage regarding the 5-year overall survival [58]. Our own data emphasize the need for multimodal approaches, as surgery alone is unlikely to improve the outcome of patients with intrahepatic CCA [53].

### The role of gut microbiota in CCA

Hippocrates of Kos, an ancient Greek physician, is said to have stated 2500 years ago that all diseases begin in the gut [59]. Unlike the other organs, the liver and the bile ducts have a special relationship with the gut and its inhabitants, the gut microbiota. The interaction between the intestine and the liver may also be crucial for the development and the progression of CCA, which will be a focus of the next paragraphs.

### The “gut-liver axis” and its particularities

Due to the close relationship between the liver and the intestine, the concept of a “gut-liver axis” has become more and more popular in recent years [60]. The “gut-liver axis” implies that the gut can influence health and disease of liver and vice versa. Both the intestine and the liver have challenging tasks, as they have to absorb nutrients and useful molecules and, at the same time, filter and excrete potential harmful substances [61]. Environmental toxins can also enter the body via the gut-liver axis [62].

The human body, especially the gut, is home to billions of microorganisms that are constantly in contact with its bodily functions, and influence the immune system’s response [63, 64]. In a healthy gut, the intestinal barrier helps to maintain the stability of the microbial colonization and to prevent the uncontrolled absorption of molecules of all kinds, regardless of whether they are ingested or produced by local microorganisms. The gut barrier consists of a physical barrier, a single layer of columnar epithelium, an inner and outer mucus layer and of several immune cells, secretory immunoglobulins and antimicrobial peptides [60]. The intestinal barrier is dynamic and can adapt to the needs of the body, but it can also be disrupted by lifestyle factors, age and a variety of diseases [59, 65]. Disruption of the intestinal barrier is associated with the onset of gut diseases like inflammatory bowel disease [66]. Interestingly, several studies also emphasize the role of dysbiotic gut microbiota in liver diseases such as nonalcoholic fatty liver disease (NAFLD) [67]. It is assumed that the disturbed gut barrier in NAFLD leads to an increase in endotoxins and lipopolysaccharides entering the portal vein. As early as 1977, Lumdsen et al. were able to demonstrate a gut microbiota-derived endotoxemia of the hepatic vein and the portal vein in patients with liver cirrhosis [68]. Henao-Mejia et al. reported that microbial substances activate toll-like receptors, which results in the release of interleukins and promotes non-specific inflammation of the liver [69]. Furthermore, gut microbiota can produce potentially harmful substances such as alcohol, which accelerate the development of liver disease and aggravate its course [64, 70]. Microbial changes associated with the severity of fibrosis are found in patients with liver fibrosis, but the liver fibrosis itself leads to changes in the microorganisms’ environment, which affects in turn the microbial composition [61]. Therefore, the “gut-liver axis” seems to be a vicious circle in the development of liver disease. Interestingly, Yin et al. stated that the gut microbiota has an influence on the regenerative capacity of the liver via the gut-liver axis. The authors investigated the regenerative capacity of the liver and found that antibiotic treatment impaired the regenerative capacity of the liver [71]. This emphasizes the possible relevance of the gut microbiota for liver health.

**Fig. 2:**
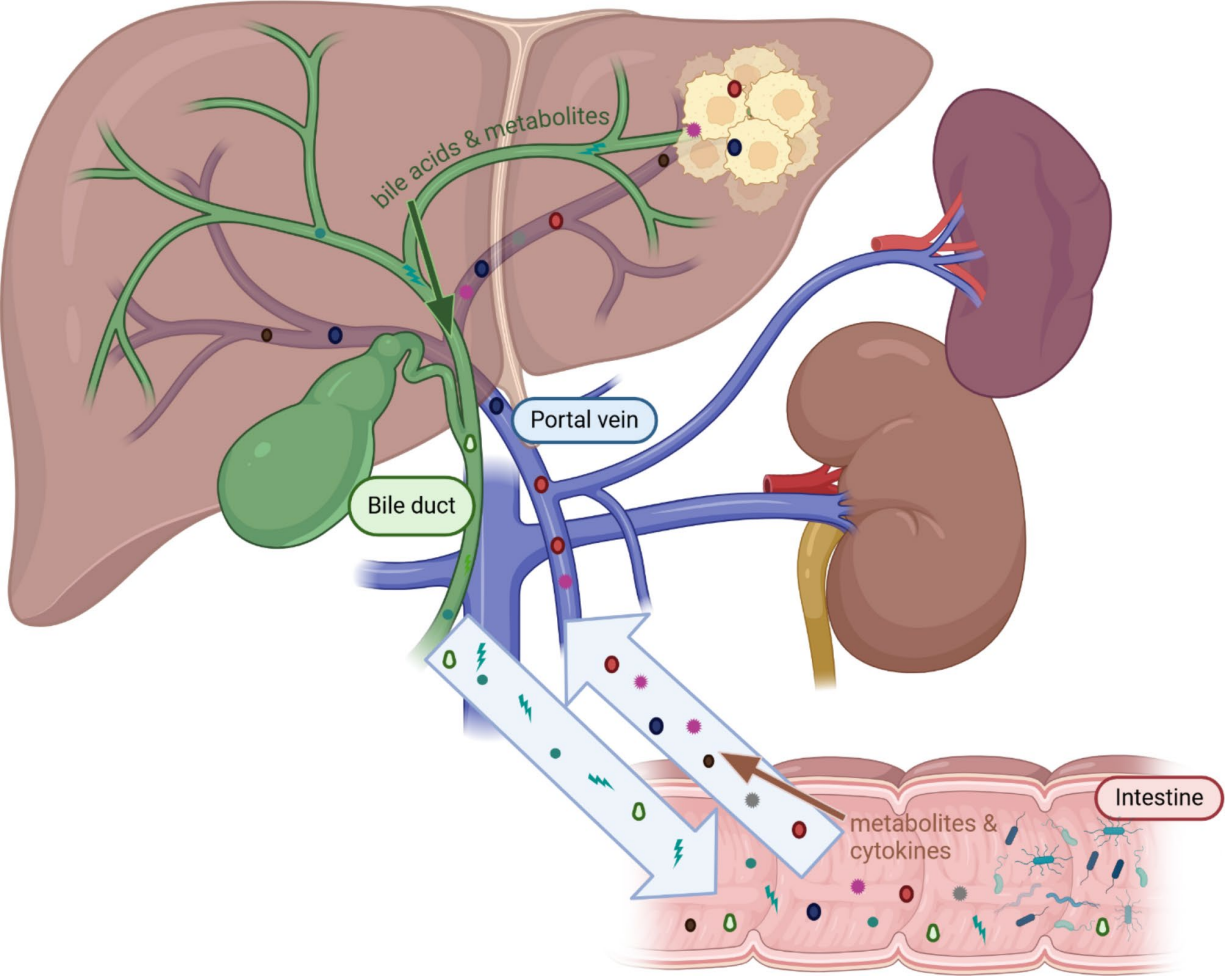
Overview of the “gut-liver axis”: a bidirectional relationship between the liver and the gut microbiota. Gut microbiota derived metabolites and cytokines enter the liver via the portal vein, biliary acids and liver-derived metabolites shape the microbial environment; created with Biorender.com

To understand the “gut-liver axis” it is essential to know the relationship between the liver and the gut. An important part of the bidirectional relationship is the so-called enterohepatic circulation, which involves the circulation of bile acids from the liver to the small intestine via the bile ducts, subsequent uptake by the enterocytes and return transport to the liver via the portal vein (Fig. 2) [72]. As mentioned before, the portal vein does not only transport bile acids, but also metabolic products, nutrients and cytokines as well as endotoxins and microbial components originating from the intestine [73]. Ingested substances and components of food enter the portal vein after they have been absorbed through the intestinal wall. Several intrinsic and extrinsic factors can affect the integrity of the gut barrier and can alter the absorption of molecules [65]. In a healthy gut, the absorption depends on the substance properties as well as on intestinal transit time and other local factors such as the pH value [72].

**Fig. 3:**
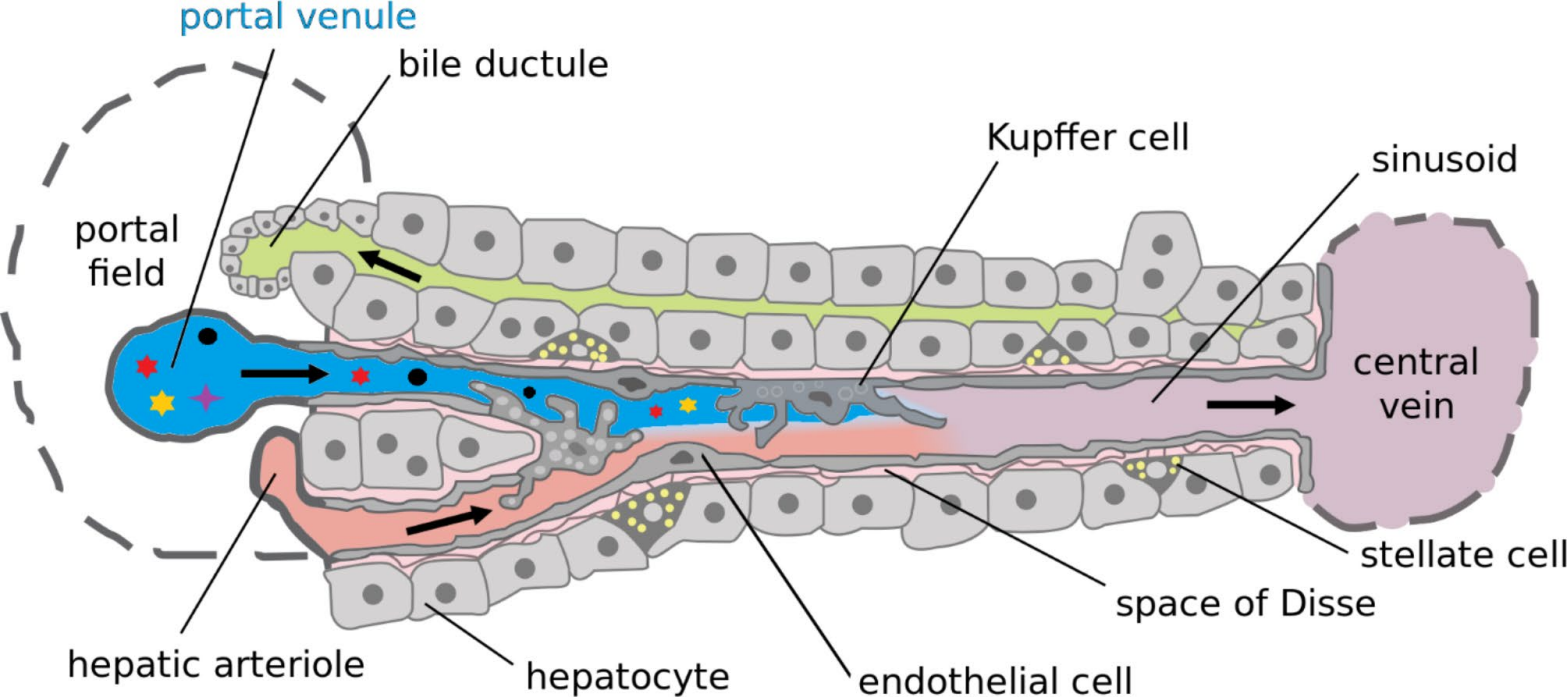
Structure of the hepatic sinusoids, a dual blood supply system that receives blood from the portal vein and from the hepatic artery. (adapted from Frevert et al. [78]). The sinusoids are lined by fenestrated endothelial cells, which facilitate the exchange between the blood and the hepatocytes

After absorption, substances and nutrients are transported to the liver via the portal vein, which divides into the left and the right portal vein and finally ramifies into the portal venules. The vessels flow into the hepatic sinusoids (Fig. 3). The hepatic sinusoids are part of a dual blood supply system, as the liver receives not only blood from the portal vein, but also from the hepatic artery, which supplies oxygen [74]. The blood flow velocity in the hepatic sinusoids is slower than in other small vessels and capillaries and is affected by the condition of the liver [75–77]. The sinusoids are lined by specialized fenestrated endothelial cells, which facilitate the exchange between the blood and the hepatocytes located on the other side of the so-called space of Disse (also known as perisinusoidal space) [77]. The microvilli of the hepatocytes protrude into the Disse space through which the blood plasma flows and contribute to the exchange of molecules.

Hepatocytes are polarized cells that have a special cellular organization ensuring their function [74, 79]. Various molecules such as nutrients but also toxins can pass through the cell membrane of hepatocytes, facilitated by different transport mechanisms. Hepatocytes are not only in contact with the blood in the hepatic sinusoids, they are also the origin of the bile ducts by forming the bile canaliculi [80, 81]. Hepatocytes excrete metabolic products and bile, which flow into the intestine via the bile ducts and shape the intestinal environment. Small molecules in particular are able to cross the hepatocytes and circulate the enterohepatic circulation [82]. However, the endothelial cells form a barrier, which can protect the liver from damage by absorbed substances. Thus, damage to the intestinal barrier does not necessarily lead to damage in the liver and the bile ducts, but it is plausible, as most diseases associated with a disturbed intestinal barrier are systemic diseases that also lead to a disruption of the liver barrier [83].

### Microorganisms in the biliary system

In addition to molecules that end up in the bile ducts via the enterohepatic circulation, as explained above, the bile ducts themselves can also be colonized with microorganisms. Even if it is commonly claimed that the bile ducts are sterile, scientific research reveals other results [84, 85]. Cohort studies emphasize the inhabitation of the bile duct with gut microbes such as *Escherichia coli* and *Enterobacter* genera in patients without pretreatment of the bile ducts [86, 87]. Colonization of the bile ducts and the pancreatic duct can be observed more frequently in patients with biliary obstruction and also after endoscopic retrograde cholangiopancreatography (ERCP) [88, 89]. The impact of a microbial colonization of the bile ducts for the development of diseases in otherwise healthy subjects is unknown. The microbiological examination of the bile ducts is invasive and involves manipulation of the bile ducts or even surgery, which makes clinical studies in healthy subjects almost impossible. In patients with benign or malignant obstructions of the biliary tract who require biliary stenting, typical inhabitants of the gastrointestinal tract such as *Enterococcus spp.*,* Enterobacteriacea spp.* or *Candida spp.* are usually found in the biliary tract [90]. In patients with PSC, the colonization of the bile ducts with *Enterococcus spp*. or *Candida spp.* leads to a disease progression [91]. Di Carlo et al. showed that *Escherichia coli* and *Pseudomonas spp.* can be found in the bile of patients with pancreatic carcinoma or distal bile duct carcinoma [92]. Bednarsch et al. reported that the majority of patients with a perihilar CCA, who underwent curative surgery, had microbial colonization of the bile ducts, predominantly with typical inhabitants of the gastrointestinal tract. The morbidity of patients was comparable in both groups (colonized vs. non-colonized), but patients with bacterial colonization had a higher mortality rate [93]. Another study by Cammann et al. showed that the bile ducts of more than 80% of patients with extrahepatic CCA were colonized with bacteria, often with antibiotic-resistant bacteria that worsened patients’ outcome [94]. In 2016, Costi et al. reported a worse outcome of patients with *Escherichia coli* bile colonization who underwent pancreatic surgery [95].

The colonization of the bile ducts by intestinal inhabitants can cause serious problems. The impaired bile flow caused by the tumor-related bile duct obstruction can lead to cholestasis, which can ultimately lead to cholangitis in the case of bacterial colonization. Cholangitis can be a life-threatening problem that is often associated with high fever, pain and a disturbed general condition and can easily proceed to sepsis [96, 97]. The occurrence of cholangitis can delay the necessary therapy, as neither surgical therapy nor chemotherapy are possible in acute infection [97, 98]. It remains unclear to what extent bile duct colonization could influence the oncological outcome of affects patients. For the gut microbiota three mechanisms are described, which potentially foster the development and the progression of cancer: first, bacterial metabolites and toxins; second, modulation of the host’s local and systemic immune response; third, metabolic changes of the microbiota and of the host [99]. Of course, all these factors also apply in the case of bile duct colonization, but there is lack of evidence for the link between bile duct colonization and a worsening of oncological outcome. Besides microorganisms in the biliary tract, there is also evidence of microbes that reside within the tumor [100, 101]. Chai et al. investigated intrahepatic CCA and found gut microbes, but also environmental bacteria such as *Paraburkholderia fungorum*, which could influence immune interactions and tumor growth [102]. Recent research indicates that CCA are surrounded by cells such as cancer-associated fibroblasts with immunosuppressive functions, which can lead to an imbalance of the local immune system and foster tumor progression [103, 104]. The whole tumor microenvironment is the focus of current research, since cellular interactions, including those with microorganisms, can contribute to tumor cell growth and susceptibility to therapies.

### Gut microbiota in CCA

First of all, it should be noted that patients with CCA are often treated with antibiotics, e.g. for cholangitis, which leads to an alteration of the gut microbiota. Therefore, many CCA patients have an iatrogenic selection of gastrointestinal microorganisms, which makes the assessment of a potential influence of the patient’s own microbiota much more difficult or even impossible. Regardless of where a change comes from, it is still necessary to evaluate the influence of the current microbial composition. The above mentioned plausibility of a relationship between the gut microbiota and CCA via the gut-liver axis is strengthened by observations in hepatocellular carcinoma (HCC), another much more common primary liver tumor. The potential mechanism of gut microbiota contributing to carcinogenesis might be similar, as HCC originates from hepatocytes adjacent to cholangiocytes (Fig. 3). In 2012, Dapito et al. demonstrated the relation between the gut microbiota and the Toll-like receptor (TLR)4-dependend promotion of HCC in an animal experiment. Interestingly, the gut sterilization with ampicillin, neomycin, metronidazole and vancomycin in drinking water decreased the promotion of HCC [105]. A few years later, it was stated that the composition of the gut microbiota is able to influence the development of HCC in patients with liver cirrhosis [106, 107].

Several reviews discuss the role of gut microbiota in CCA development and progression [108, 109]. A previously published scoping review of our group revealed inhomogeneous microbial changes in CCA patients compared to healthy controls [4]. Overall, the results were not conclusive due to methodological shortcomings such as insufficient information on antibiotic treatments and lack of consistency throughout the studies. Genera that were observed less frequently in CCA patients compared to healthy controls were, for example, *Faecalibacterium* and *Ruminococcus*. The decrease in *Faecalibacterium*, a genus often discussed as a health marker due to its anti-inflammatory activity, seems plausible in the case of cancer [110]. However, the decrease of *Ruminococcus* is not easy to interpret as *Ruminococcus spp.* is associated with both positive and negative effects [111, 112]. Other gut microbiota changes might be a reflection of the clinical condition of CCA patients as *Megamonas* is known to be decreased in patients suffering from tumor cachexia [113].

The scoping review also focused on the question of the extent to which the gut microbiota influences the prognosis of patients with CCA, but could not provide a clear answer to this question either. Jia et al. reported higher abundance of the family *Oscillospiraceae* and lower abundance of the family *Eubacteriaceae* as well as of the genera *Allobaculum*, *Pediococcus*, *Pseudoramibacter*, and *Peptostreptococcus* in CCA patients with venous infiltration compared to CCA patients without venous infiltration [114]. Vascular infiltration is known to worsen the outcome of CCA patients [115, 116]. Zhang et al. showed distinct effects of the gut mycobiota composition of patients with advanced stage CCA (stage III and IV) compared to CCA patients with lower stages (stages I and II) emphasizing a potential role of *Candida albicans* in advanced disease [117]. Mao et al. reported that the order *Bacteroidales* was positively associated and the family *Veillonellaceae* was negatively associated with progression-free survival and overall survival [118]. An animal trial by Zhang et al. emphasized the role of gram-negative bacteria in CCA progression of patients suffering from PSC [119]. Two recent studies focused on the composition of the gut microbiota and the response to treatment with programmed cell death receptor-1 (PD-1) antagonists in patients with advanced CCA. Both publications report distinct, but also different changes in the gut microbiota, which are said to have had an influence on the progress of the included patients [118, 120]. The impact of the gut microbiota on the efficacy of systemic drug therapy for cancer, especially with newer substances such as PD-1 antagonist, is a subject of ongoing debate [121].

Nevertheless, the difficulties outlined emphasize the challenge of clinical microbiota research. The individuality of each patient makes it difficult to compare them with other patients, even if they have the same disease [1]. Moreover, CCA are, as explained in the beginning, a particularly inhomogeneous group of tumors, which makes clinical microbiota research even more challenging.

### Cell culture fundamentals

At the beginning of the 20th century, many scientists such as Wilhelm Roux, Franklin P. Mall, Montrose Thomas Burrows and Ross Granville Harrison have contributed to the development of today’s cultivation of cells by their animal and cell experiments [122, 123]. Alexis Carrel was one of the first to describe the successful in vitro cultivation of malignant cells more than 100 years ago [124]. Meanwhile, the cultivation of human, animal or plant cells in a nutrient medium outside their natural environment has been an essential part of scientific research for decades. Cell cultures are in vitro models living in a modifiable and controlled condition [125]. They offer the possibility of observing the behavior and growth of cells under self-selected conditions. Cell cultures can not only simulate in vivo situations, but can also be exposed to conditions that could be harmful for the donor organism, which is why they can contribute to a better understanding of the metabolism and physiology of cells, even in critical and life-threatening situations [125].

### Cell lines and their cultivation

Cell cultures must be grown in sterile conditions, as otherwise they may be contaminated with microorganisms that impair cell growth and function [126, 127]. In order to grow, cells need a suitable medium and the appropriate growth factors and nutrients. To produce a cell culture, the tissue or cells of interest can be obtained from a human or animal donor organism (so-called primary cells). The isolation of cells capable of growing in a cell culture requires enzymatic or mechanical extraction methods [128]. The cultivation could be challenging depending on the tissue from which the cells originate. Most cells, especially primary cells, are sensitive to their environment, and must be cultivated at body temperature [126]. Primary cells are non-immortalized cells that are subject to an aging process (“senescence”) and usually die after a few weeks to months [128]. In vivo, the cellular senescence is a dynamic process that can be influenced by several internal and external factors and appears to play an important role in the cellular network [129, 130]. Senescence can be used in vitro to explore the effects of (cellular) aging [131]. Primary cells offer the advantage of a direct connection to the donor organism, which is in line with the increasingly important principles of personalized medicine. Thus, primary cells reflect the metabolism and the characteristics of the donor organism and are intended to be an in vitro model of the donor organism.

Alternatively, cells from an immortalized cell line can be used for cultivation. These cells can be obtained from cell banks such as the German Leibniz Institute DSMZ [132]. Immortalized cells are able to overcome the cell division limit and continue to divide due to a natural or experimentally induced mutation. Immortality can be achieved, if cell cycle checkpoints are deactivated [133]. Immortalized cells have little relation to the cell metabolism of healthy organisms, but contribute to the understanding of malignancy and toxicity. Immortalized cell lines can be used, for example, to produce proteins and growth factors for medicinal purposes and research [128]. A very well-known example of an immortalized cell line are the cells of Henrietta Lacks, who suffered from an aggressive adenocarcinoma of the cervix in the early 1950s and whose cells are still used in research today as HeLa cells [134]. HeLa cells outlived their donor Henrietta Lacks by many decades as she died on October 4, 1951. Common examples of immortalized human CCA cell lines are HuCCT-1 cells or RMCCA-1 cells [135, 136].

### The classical two-dimensional cell culture

**Fig. 4:**
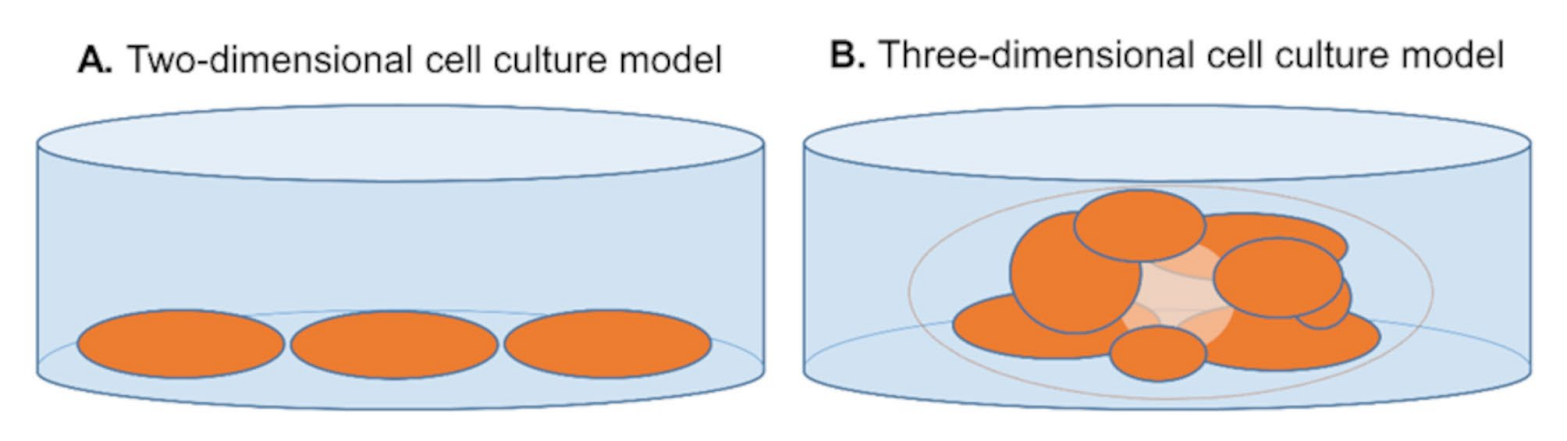
Two-dimensional (**A**) and three-dimensional (**B**) cell culture models. The three-dimensional model grows in a matrix (gray border) that prevents contact between the cells and the dish and supports spatial organization of the cells to form 3D structure

Today, the classical and predominant cell culture is a two-dimensional (2D) cell culture, which plays an essential role in diagnosis, prognosis and treatment of several diseases such as cancer [128]. Cells in a 2D cell culture grow side-by-side as a monolayer implying little cell interaction and differentiation (Fig. 4A) [137].

The design of the cell culture allows a homogeneous supply of growth factors and nutrients to all cells, which is why all cells show the same growth behavior and morphology [138, 139]. Classic 2D cell cultures consists of only one cell type, which is fixed in a defined condition through cultivation [140]. Therefore, the major disadvantage of 2D cell models is the lack of interaction between the cells and the fixation. Growth in a 2D cell culture restricts the cells in their morphology, their cellular behavior and their expression of surface receptors [138]. Epithelial cells, for example, are influenced by the properties of their environment, so that they can take on a different morphology in cell cultures [141]. In vivo, cells grow in associations of different cells and communicate with each other and with their environment. Classic 2D cell cultures are not able to mimic interactions between cells and, of course, also with matrices and other body components [139]. Especially for cells that actually live in a complex system such as the cells of the gastrointestinal tract, the results of 2D cell models should be evaluated critically [140]. This problem is also reflected in the results of experiments, as cells in 2D cultures show a different behavior than cells in more complex cell models or in the human body [142]. Although there are models designed to help reduce the fixation of the cells, they involve considerable effort and are not suitable for every situation [143, 144].

Sandwich cell cultures and cell co-culture models can be used to address the lack of interaction with the immune system and the surrounding microenvironment [145]. For this purpose, different cells are cultured together or in close proximity in order to investigate direct cell interactions or interactions through messenger substances. If only the cell interaction via messenger substances is planned to be investigated, the supernatant of cells can also be given to the cells of interest. However, it remains a problem that even co-cultured cells grow in a 2D cell culture that does not correspond to their natural environment. The cell fixation and the unnatural growth of cells in 2D cell culture is why more complex models are needed. The altered growth behavior and the cell morphology in 2D cell cultures, which differs from that of in vivo cells, can otherwise influence the results of investigations [138, 142].

### Organoids: a three-dimensional phenotypic cell culture

Interestingly, scientists have been working on more complex cellular models for more than 100 years [146]. One of the first scientists, who described the capability of whole organism regeneration of dissociated sponge cells was Henry Van Peters Wilson in 1907 [147]. He was able to show that it is possible to renew a complex cell system through single cells. The aim of complex cell models such as the three-dimensional (3D) cell culture is to allow the cells to grow more naturally and to interact with others cells and their environment. The advantage of more natural growth behavior and morphology of cells has led to 3D cell models such as the “organoid” model becoming increasingly important in the scientific world. Even if mouse models are still a gold standard for many research questions, they are not transferable to humans in every respect and are more time-consuming than organoid models [148]. There are various animal models for CCA, with xenografts being among the most widely used in cancer research [149]. Xenografts involve transferring cells or tissue from one species to another, such as implanting human CCA cells into mice [150]. This allows the tumor to interact with a living organism, mimicking cellular dynamics. However, since the host organism is not human and its immune system must be suppressed to allow tumor growth, the comparability with real cancer patients is limited [151]. Kim et al. reported in 2020 that organoid models are superior to other model systems because they are easy to establish and maintain, offer a wide range of possibilities and recapitulate human physiology [152]. Nowadays, 3D cell models are able to imitate physiological and pathological processes in the human body and thus contribute to a better understanding of health and disease [153]. It is therefore not surprising that the US Food and Drug Administration (FDA) has cleared the way for avoidance of animal testing in pharmacological studies and the use of new methods such as 3D cell culture models [154].

The growing of 3D cell models is technically similar to the growth of 2D cell culture, but the originating cell is different. The potential of the organoid model is already evident here, as no mature cells are cultivated, but rather stem cells, which have a different biological potential [155]. Organoids can be grown from different types of stem cells, either isolated adult stem cells from donor tissue (similar to primary cells in 2D cell cultures), embryonic stem cells or induced pluripotent stem cells (iPSC) [156, 157]. The technology of iPSC was first introduced in 2006 by Shinya Yamanaka, who was later awarded the Nobel Prize for his discovery. Mature cells can be reprogrammed into pluripotent stem cells by introducing the so-called Yamanaka factors (four specific genes) [158]. The so produced iPSCs can be used not only to grow organoids, but also for tissue repair and whole organ regeneration [159]. In contrast to a classical 2D cell culture, all stages of cell cycles and cell life can be found in organoids: proliferating cells, resting cells, apoptotic or necrotic cells, small or large cells, older and younger cells [138]. Organoids have the same tissue architecture as the donor tissue and contain not only differentiated cells but also stem cells [160]. Cancer organoids can be grown from tumor samples and are capable of mapping cellular characteristics as well as the phenotype and the genotype of the donor tumor tissue [161–163]. These organoids are also called “tumoroids” [164]. Some publications indicate that tumoroids can be kept in culture for months to years without a significant change of their morphology or their cellular characteristics [165, 166]. Interestingly, in contrast to immortalized cells in 2D cell cultures, it appears that they do not lose their physiological cellular behavior and growth during cultivation [146].

For organoid cultivation, individual cells must be isolated by enzymatic dissociation from the tissue. The isolated tumor cells (and also other cells such as the iPSC) can be seeded after further washing and processing [156, 165]. Unlike 2D cell models, 3D cell models do not grow as a monolayer on glass or plastic, but in a biologically derived or synthetic-based extracellular matrix (Fig. 4B). In general, it is desirable that there is no direct contact between the cells and the dish, which is why a scaffold is used. There are also scaffold-free approaches like the “Hanging-Drop Method” [167]. Current publications usually report the use of a matrix as scaffold, a composition of adhesive proteins such as collagen, entactin, laminin, and heparin sulfate proteoglycans that mimic an extracellular matrix [146]. The matrix allows an exchange between the cells and freer cell growth. Thus, the cells can interact with each other, but also with the extracellular matrix and the microenvironment, which corresponds more to the real growth conditions. Not surprisingly, the growing of primary 3D cell cultures has similar challenges as the growing of primary 2D cell cultures as cells are sensitive to their environment [126]. Organoid cultures require a tissue-specific medium that contains various growth factors and signaling proteins such as epidermal growth factor (EGF), R-spondin (RSPO)1, Noggin and wingless-related integration site (Wnt)-3a [156, 165, 166]. In tumoroids, it may be necessary to adapt the composition of the medium to the genetic characteristics and requirements of the donor tissue in order to enable optimal growth of the tumoroids [168]. The mutation of driver genes contributes to the fact that it is possible to have tumoroids in culture for months to years, as already mentioned in the beginning [165, 166]. Similar to 2D cell cultures, it is also possible to create organoid co-cultures, e.g. with immune cells. Complex organoid co-cultures are able to mimic essential parts of the immune system and of the tumor microenvironment [160]. For research questions, it is also possible to affect and control the growth and the morphology of organoids by adding substances of interest to the medium. It is also assumable that luminal spaces of organoids can be filled with substances. This mechanism of inoculation ideally requires a micro- or nanoinjector with which the substance of interest can be injected into the interior of the organoid [169, 170].

The growing of organoids, especially tumor organoids, requires experience and patience, as some cell types only show their three-dimensionality after days to weeks and grow slowly [171]. Jarno Drost and Hans Clevers stated that tumoroids do not necessarily grow faster than normal tissue, as they might be more susceptible to mitotic failure and subsequent cell death due to genetic defects [148]. The success rates for growing organoids from donor tissue vary in literature. In personal scientific discussions, it is often reported that organoid cultivation is difficult, but recent literature suggests that success rates of up to 90% can be achieved depending on the donor tissue, the experience of the scientist and the technique used [171, 172]. The success rate for growing CCA organoids appears to be lower. Saito et al. reported a success rate for the growing of patient-derived CCA organoids of around 50% [162]. Maier et al. reported that they were initially unable to grow CCA organoids for more than two passages, but a modification of the medium made the growth possible [150]. From our own experience, we would agree that it is difficult to achieve a similarly high success rate of 90% as for colorectal carcinoma organoids in CCA organoids. From a clinical perspective, one factor contributing to a lower success rate is the fact that perihilar and distal CCAs can be small tumors, resulting in an insufficient amount of tumor cells for organoid cultivation.

### Critical view and limits of organoid technique

**Fig. 5:**
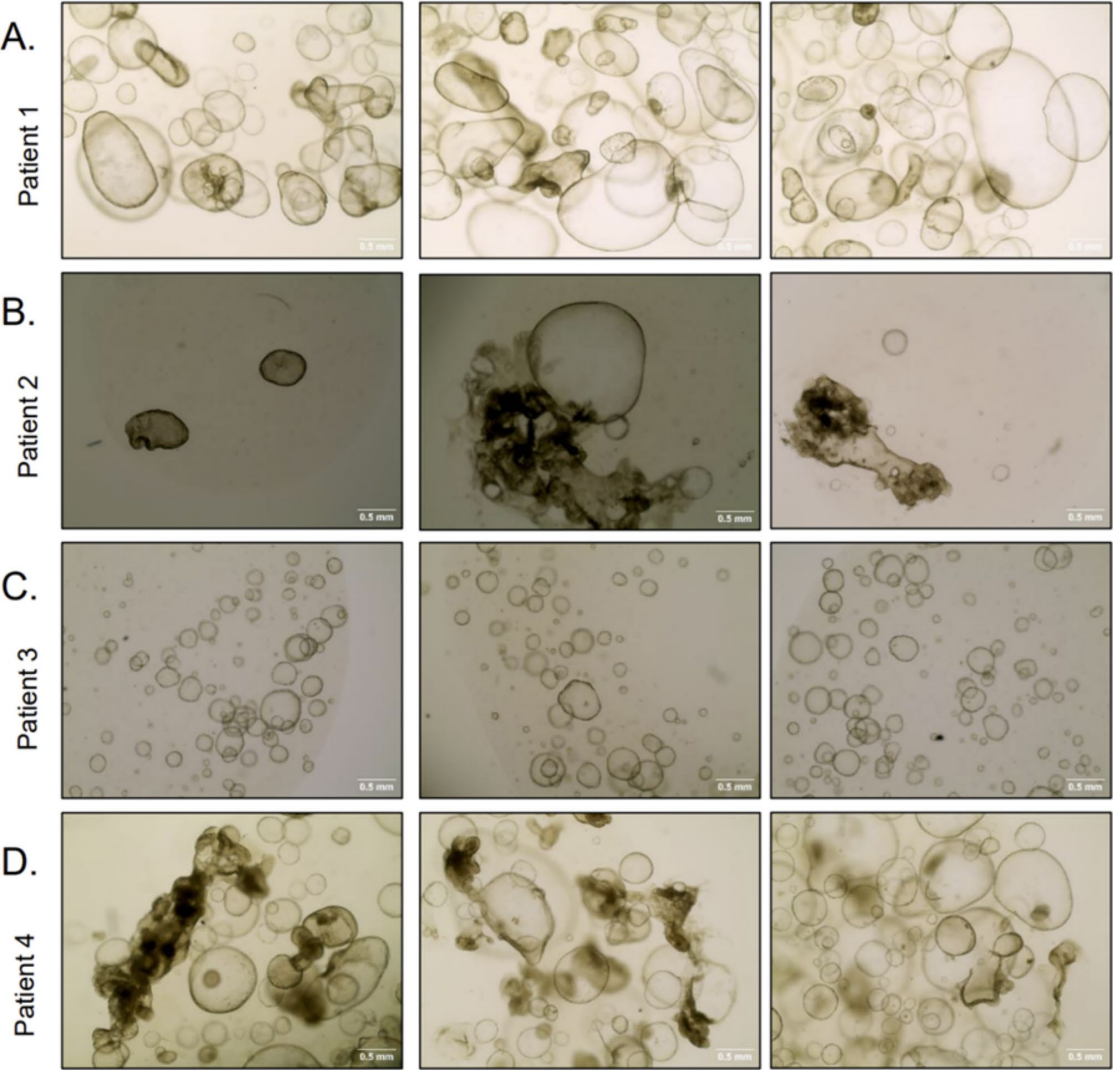
Microscopic impressions of the morphology of patient-derived cholangiocarcinoma organoids from four different patients (**A**, **B**, **C** and **D**) after four weeks of cultivation and four passages. Although all organoids were captured at the same magnification, there are clear differences in size, morphology, density and sphericity

Patient-derived organoids show differences in their growth behavior and their morphology, which might be a consequence of cellular characteristics and genotype differences of the donor tissue [171, 173]. Organoids offer the advantage of being an individualized tumor model of the patient, which can be used to test the efficacy of drug therapies. Organoids are therefore a valuable tool for precision medicine [173–175]. The differences in growth behavior and morphology are also evident in CCA organoids (Fig. 5).

CCAs are a very heterogeneous group of tumors, which makes organoids all the more interesting. Cho et al. demonstrated that the ability of organoids to reflect the subtypes of intrahepatic cholangiocarcinomas can be used to test individualized drug therapies preclinically [176]. Rimland et al. demonstrated differences in tumor characteristics based on their location within the biliary tract by using organoids. The authors were able to show that organoids derived from extrahepatic and intrahepatic bile ducts exhibit distinct growth requirements, reflecting their site-specific biology. Transcriptomic analyses further revealed significant differences between these organoids, despite all originating from the biliary tract [177]. Thus, the differences between patient-derived organoids reflect the heterogeneity of CCAs and open up the possibility of gaining a better understanding of an individual’s CCA. This is an example of an advantage over immortalized CCA cell lines such as HuCCT-1, which do not exhibit individual characteristics. While the variability of organoids complicates direct comparisons, this same feature mirrors the real-world heterogeneity of CCAs, making organoids instrumental in advancing personalized medicine and understanding tumor dynamics in a biologically relevant context, as cell growth and behavior also differ between patients in vivo. In 2020, Pleguezuelos-Manzano et al. emphasized the individuality of each patient-derived organoid [171].

A general problem with cultivation of organoids from donor tumor cells may be that not only tumor cells grow in the culture, but also healthy neighboring tissue. In Fig. 5, it is not possible to tell from the morphology of each individual cell whether it is actually malignant. Both malignant and benign cells are found in organoid cultures, which differ in their growth pattern but cannot be distinguished with the naked eye [165]. Indeed, the growth of malignant and benign cells side by side is not necessarily a disadvantage of a 3D tumor cell culture, as this reflects the reality in the human body and allows the tumor cells to grow in a natural environment. Kinoshita et al. reported that after the third to fifth passage, the presence of non-cancerous bile-derived organoids decreases, whereas the cancerous organoids remain [178]. In addition to the evaluation of organoid morphology, immunohistochemical markers or invasion assays can be used for differentiation of cells [179]. If necessary, genetic analysis can help to characterize and identify the cell types [156]. However, additional experiments can be expensive, which is a problem of organoid growing anyway. Reagents and growth factors used for organoid culture can be very expensive, especially when compared to classical 2D cell cultures [152]. Scientist are discussing the extent to which the reagents used can be reduced without jeopardizing the results of the organoids [173]. To reduce costs, immortalized cell lines can be used to produce otherwise expensive growth factors such as Wnt3a.

As already introduced in the previous section, it can be difficult to successfully grow organoids. The ability of organoid growth depends on the quality of the donor tissue. In tumors, there are different areas in which vital cells, necrotic cells or even tissue fibrosis can be found [180]. Furthermore, cells can lose their specific phenotypic characteristics during cultivation. This process is called “dedifferentiation”, and it is problematic as dedifferentiated cells are no longer a model of the donor organism. This condition can be prevented by treating cells with a medium and conditions that are optimal for them [125]. Uematsu et al. have shown that the dedifferentiation of breast cancer cells that lose estrogen receptor expression and transition from a luminal to a basal breast cancer after passage can be counteracted by inhibiting NOTCH signaling [181]. However, when cultivating tumor tissue, it may not always be clear which conditions are optimal for tumor growth as mutations could contribute to changes in growth requirements. The observation by Uematsu et al. emphasizes that a deeper understanding of the molecular processes is necessary to be able to work successfully with cancer organoids. The complexity of organoid models can complicate the analysis of results. For example, image analysis of organoids can be challenging because organoids grow in a three-dimensional space and therefore show overlapping of cells (Fig. 4). Programs such as Cellos and MOrgAna can help with the analysis of organoid images [182, 183].

*What to expect: understanding the “gut-liver-axis” and the role of gut microbiota in a patient-derived CCA organoid model*.

Organoids are a future research concept, but their potential in the context of studying the “gut-liver axis” has only been partially exploited. Organoids have been used in recent studies to understand the development and progression of congenital and acquired liver diseases [184]. Ouchi et al. reported the results of multicellular liver organoids incubated with fatty acids [185]. Fatty acids are not only a source of energy for the human body, but also a signaling molecule. Fatty acids are absorbed into the body both through food intake and through some type of intestinal bacteria, which produce for example short-chain fatty acids when fermenting fiber [186]. Interestingly, the treatment with fatty acids led to an increase of organoid stiffness, reflecting the progression of liver fibrosis [185]. In another study, Ramli et al. were able to show that liver organoids had the same genetic signature as nonalcoholic steatohepatitis (NASH) liver tissue after treatment with fatty acids [187]. These two studies emphasize that organoid models can also be used to investigate the effect of food components or gut-derived bacterial metabolites on the human body. Furthermore, co-cultures offer broad possibilities to better understand the relationship between nutrition, microbiota and tumor growth by simulating various interacting cellular models. De Crignis et al. demonstrated in an organoid model to study hepatitis B and the subsequent development of hepatocellular carcinoma that these organoids are both suitable for studying therapy-induced toxicity and show an early cancer-related gene signature [188]. The results of these studies emphasize the ability of organoids to act as a cellular model for liver diseases. In 2017, Sampaziotis et al. indicated a groundbreaking idea regarding the biliary tract. The authors stated that regeneration of the bile ducts could be possible with the help of organoids as organoids were able to repair surgical defects of the gallbladder wall and replace damaged bile ducts [189]. Organoids could therefore not only be cellular models of the biliary tract, but also a therapeutic approach in case of damaged bile ducts.

To date, only a handful of research groups have already reported on the growth of CCA organoids, but there are no data on patient-derived organoid models regarding the relationship between the gut microbiota and the progression of CCA [150, 162, 190]. However, there are several studies that have examined the effects of components of the gut microbiota on intestinal organoids. Kadosh et al. reported that p53 mutant jejunum organoids showed a rounding of organoids after treatment with polyphenols [191]. The treatment with gallic acid, a food-derived polyphenol, led to a suppression of the WNT activity, which is typically activated in cancer [191, 192]. As mentioned earlier, this study also emphasizes the potential of organoids to investigate nutrition-related effects on tumor growth. Another study by Freire et al. examined the effects of the microbiota in coeliac disease. The authors treated human celiac disease organoids for 48 h with butyrate, lactate and polysaccharide A extracted from *Bacteroides fragilis*. All substances showed an anti-inflammatory effect in case of contact with gliadin and ameliorated changes of the transepithelial electrical resistance [193]. Sugimura et al. incubated colorectal cancer organoids with the supernatant of *Lactobacillus gallinarum* and *Escherichia coli*. Interestingly, the incubation with the supernatant of *Lactobacillus gallinarum* led to a concentration-depended suppression of cell proliferation [194]. However, it must always be borne in mind that microbial components are actually found intraluminally in the living organism. It may therefore be important that organoids are not only treated with bacterial components, but that the luminal spaces of the organoids are also filled with these. In summary, all of these studies indicate the changeability of organoid systems with regard to their morphology and growth behavior after treatment with gut-derived and gut microbiota-related substances. Organoids could therefore be a promising approach to get a deeper understanding of the “gut-liver axis” and its functionality. A patient-derived organoid model could contribute to a better understanding of the role of the gut microbiota in CCA progression, which could also help to improve future treatment strategies. By applying machine learning models, individualized organoid data could potentially help predict the therapeutic success of an individual patient [195]. A multidisciplinary approach is essential to fully understand and address the complexity of CCA. This approach should integrate not only clinicians but also computational biologists, geneticists, microbiologist, and experts from diverse fields, fostering collaborative research to uncover new therapeutic strategies and biomarkers for personalized treatment.

## Conclusions

The CCA is an aggressive primary liver tumor that is associated with a high recurrence rate and a poor prognosis for affected patients. Despite scientific advances in recent decades, the prognosis of CCA has changed very little. There is a need to develop new research models to identify potentially modifiable factors that influence the progression and prognosis of CCA. The liver and bile ducts have a close bidirectional relationship with the gut microbiota. Recent research findings suggest that the gut microbiota may not only have an influence on the progression of cancer, but also on the response to drug therapies. Therefore, a promising approach that could improve treatment strategies for CCA would be a deeper understanding of the importance of this so-called “gut-liver axis”. The investigation of the gut microbiota in cancer progression and prognosis by clinical research is challenging due to a variety of potential biases. Thus, the use of complex cell cultures such as organoids and organoid co-cultures might be powerful and valuable tools to study not only the growth behavior and growth of cells, but also the interaction with the tumor microenvironment and with components of the gut microbiota. Future research should therefore take advantage of organoid models to better understand the “gut-liver axis” and derive consequences for better treatment of patients with CCA.

## Data Availability

We do not analyze or generate any datasets due to the nature of a review.

## Abbreviations

ALLPS: Associating Liver Partition and Portal vein ligation for Staged hepatectomy
CCA: Cholangiocarcinoma
EGF: Epidermal growth factor
EGFR: Epidermal growth factor receptor
ERCP: Endoscopic retrograde cholangiopancreatography
FDA: US Food and Drug Administration
FGFR: Fibroblast growth factors
FLR: Futures liver remnant
HCC: Hepatocellular carcinoma
IDH: Isocitrate dehydrogenase
iPSC: Induced pluripotent stem cells
KRAS: Kirsten rat sarcoma virus
NAFLD: Nonalcoholic fatty liver disease
NASH: Nonalcoholic steatohepatitis
PD-1: Programmed cell death receptor-1
PSC: Primary sclerosing cholangitis
p53: Transformation-related protein 53
RSPO: R-spondin
TLR: Toll-like receptor
TRM: Tumor-resident microbes
WNT: Wingless-related integration site
2D: Two-dimensional
3D: Three-dimensional
